# Improving the Accuracy of the Gynaecology Handover Process: An Effective Quality Improvement Project at a University Hospital in the United Kingdom

**DOI:** 10.7759/cureus.68889

**Published:** 2024-09-07

**Authors:** Indranil Banerjee, Gargi Mukherjee, Sujatha Kalburgi, Abhyuday Chanda

**Affiliations:** 1 Obstetrics and Gynaecology, Oxford University Hospitals NHS Foundation Trust, Oxford, GBR; 2 Obstetrics and Gynaecology, Medway Maritime Hospital, Medway NHS Foundation Trust, Gillingham, GBR; 3 Obstetrics and Gynaecology, Basildon University Hospital, Mid and South Essex NHS Foundation Trust, Basildon, GBR; 4 Biostatistics, Benaras Hindu University, Varanasi, IND

**Keywords:** surgical handover, sbar tool, patient safety culture, gynaecology and obstetrics, quality improvement projects, safe patient handover

## Abstract

Objectives

The objective of this study was to introduce a new system of handover in the gynaecology department and ensure its effectiveness with dynamic improvement measures. This was launched as a quality improvement project in a district general hospital in the United Kingdom. The primary aim was to start and consolidate a new system of a separate gynaecology handover in the presence of consultants, registrars (incoming and outgoing), senior house officers (incoming and outgoing) and gynaecology nurses.

Design

The strategy for consolidation included a daily quality review on the basis of a fixed proforma, identifying the obstacles faced, and improvising dynamic solutions. A new quality check proforma was introduced which took into account: (i) Presence of team members, (ii) Following of proper SBAR (Situation, Background, Assessment, Recommendation) format in the handover, (iii) Updating of patients awaiting surgeries with every detail on the list, (iv) Proper handing over of pending referrals, (v) Mention of sick patients with proper importance, and (vi) Proper handing over of new admissions.

A pilot study was done to evaluate the baseline performance of the unit regarding the gynaecology team handover on the basis of the same proforma. The result of the baseline study was noted as the reference. Each day the team receiving the handover was interviewed for the next five months about the quality of each of the parameters on the predesigned proforma and the responses were noted. The answers were designed in binary form (Yes/No). These results were compiled at the end of each month. The result from each individual month was reviewed and the problems were identified and practical solutions were applied. These changes were noted and plotted graphically as a bar diagram. The monthly audit results were tabulated in an Excel sheet (Microsoft Corporation, Redmond, Washington, United States).

Results

Pilot study results and final month results were compared with the help of the Mcnemar test and statistically significant improvement was noticed in seven out of eleven parameters. There was a steady and gradual improvement in the responses. The possible limitations of the study were also noted at the same time.

Conclusion

The quality improvement project was highly effective in improving the quality of handover and increased patient safety to a large extent.

## Introduction

Handover forms the backbone for continuity of patient care within the National Health Service (NHS), United Kingdom. It is the exchange of information between outgoing and incoming teams. It is very much dependent on individuals and their interpretation of the clinical information. Providing a structure helps in reducing the variations in interpretation. A handover is usually defined as “the transfer of professional responsibility and accountability for the aspects of a patient's care to another person“ [1].

A structured handover process is extremely crucial in maintaining a high standard of care. It ensures that every detailed information is effectively retained and passed over to the next team. As well as improving patient safety, the handover process is important from an individual's professional perspective as well, especially in avoiding unnecessary legal issues later on [2].

An ideal handover process should abide by a few essential criteria; the manpower involved should be adequate, patients should be handed over in SBAR (Situation, Background, Assessment, and Recommendation) format, and sick patients, patients waiting for surgery/operation theatre, and pending referrals should be handed over with utmost detailing.

COVID-19 and inappropriate skill mixing have raised a huge concern over patient safety in recent years. It has even led to gynaecology patients being cared for in non-gynaecology wards by nurses with little or no formal training in gynaecology. In this scenario, the need was felt to make the handover system much more robust so that any serious incidents could be avoided. A quality improvement project was thus started which will improve the scenario to a great extent.

Prior to this project, gynaecology handovers were done in conjunction with the obstetric handover in the labour ward. The idea behind the project was to start a new system of separate gynaecology handover and make the process more robust by identifying the ongoing issues and solving them over a period of time. The SBAR is an internationally recognized format of a safe handover process, which was established via a systematic review by Muller et al. in 2018 [3].

## Materials and methods

This was a quality improvement project targeted at introducing a new system of separate gynaecology handover and improving the effectiveness of the handover process by dynamic troubleshooting methods. The greater aim was to improve the service on the aspects of patient safety and clinical effectiveness. The project was carried out in the Maternity Department, Basildon University Hospital, Basildon, United Kingdom, from January 1, 2022, to June 30, 2022 (the middle of a training year).

A pilot study was performed before the start of the project to assess the areas of improvement and the baseline data was obtained. The parameters were based on the essential guidelines outlined in NHS England Seven Day Services Clinical Standards and essentially focused on the adequacy of manpower during the handover process, whether the patients are handed over in a proper SBAR format or not, whether sick patients, patients waiting for theatre, and pending referrals are being handed over with details or not [4,5]. There were 11 parameters selected in order to evaluate the quality of the handover (Table 1). Ideally, the expected percentage should be 100%; however, as this was a quality improvement project involving human factors and a combination of multiple events, it was decided by the research team that a 90% target level would be more realistic in this case. The details of the parameters are given in Table 2.

**Table 1:** The essential parameters on the basis of which change was measured SHO: Senior House Officer; SBAR: Situation, Background, Assessment, Recommendation; MEOWS: Modified Early Obstetric Warning Score

Parameters	Measurable Variable	Expected Percentage
Team presence	Presence of consultants	90%
	Presence of day registrar	90%
	Presence of night registrar	90%
	Presence of day SHO	90%
	Presence of night SHO	90%
	Presence of nursing staff	90%
Inpatients	Percentage of inpatients handed over in SBAR format	90%
Patients waiting for theatre	Percentage of patients waiting for theatre handed over	90%
Sick/Septic patients (MEWOS score >4 or 3 in any single parameter)	Percentage of sick/septic patients handed over	90%
Pending referrals	Percentage of pending referrals handed over	90%
New admissions	Percentage of new admissions handed over	90%

**Table 2:** Details of the parameters SBAR: Situation, Background, Assessment, Recommendation; MEOWS: Modified Early Obstetric Warning Score; CEPOD: Confidential Enquiry into Peri-Operative Deaths

Parameters	Details
Team presence	Presence of the correct proportion of staff members is essential for any effective handover. Here the presence of a consultant, registrar (incoming and outgoing), senior house officer (incoming and outgoing) and nursing personnel was considered essential for the purpose. Any absence will be marked as NO.
Inpatients	According to the response noted from the incoming handover team if there are any patients which have not been handed over in the SBAR format, this parameter will be marked as NO in the daily tabulation. At the end of the month, the percentage will be calculated on the days this parameter has been marked as YES.
Patient waiting for the operation theatre	According to the response noted from the incoming handover team if there are any patients who have been waiting for theatre (for the morning slot in CEPOD) that have been missed in the handover, this parameter will be marked as NO in the daily tabulation. At the end of the month, the percentage will be calculated on the days this parameter has been marked as YES.
Sick/septic patients (MEWOS score >4 or 3 in any single parameter)	According to the response noted from the incoming handover team, if there are any patients scoring MEWOS score >4 or 3 in any single parameter has been missed in the handover, this parameter will be marked as NO in the daily tabulation. At the end of the month, the percentage will be calculated on the days this parameter has been marked as YES.
Pending referrals	According to the response noted from the incoming handover team if there are any pending referrals that have been missed in the handover, this parameter will be marked as NO in the daily tabulation. At the end of the month, the percentage will be calculated on the days this parameter has been marked as YES.
New admissions	According to the response noted from the incoming handover team if there are any new admissions that have been missed in the handover, this parameter will be marked as NO in the daily tabulation. At the end of the month, the percentage will be calculated on the days this parameter has been marked as YES.

Following the end of the pilot study, the baseline data was analyzed and the areas of improvement were identified. The entire result was dissipated among the team members in order to increase awareness about the areas of handover that were lacking effectiveness. The approach was to identify the problems and to solve them with proper utilization of the available resources. In order to measure the change in the parameters, the response from team members involved in the handover process was taken as the gold standard.

A proforma (see Appendices) was designed for this purpose containing all the parameters mentioned above and the response was to be marked in a binominal manner. The team receiving the handover was interviewed every day and the proforma was filled up according to their response. Daily data was tabulated in an Excel sheet (Microsoft Corporation, Redmond, Washington, United States) and converted to average monthly data. The monthly data was compiled over the next six months. Each month`s performance was reviewed and the problems were identified. Practical solutions were devised for the problems identified and they were applied in series. The final month’s result was compared with the pilot study results and the statistical significance was calculated.

**Figure 1:**
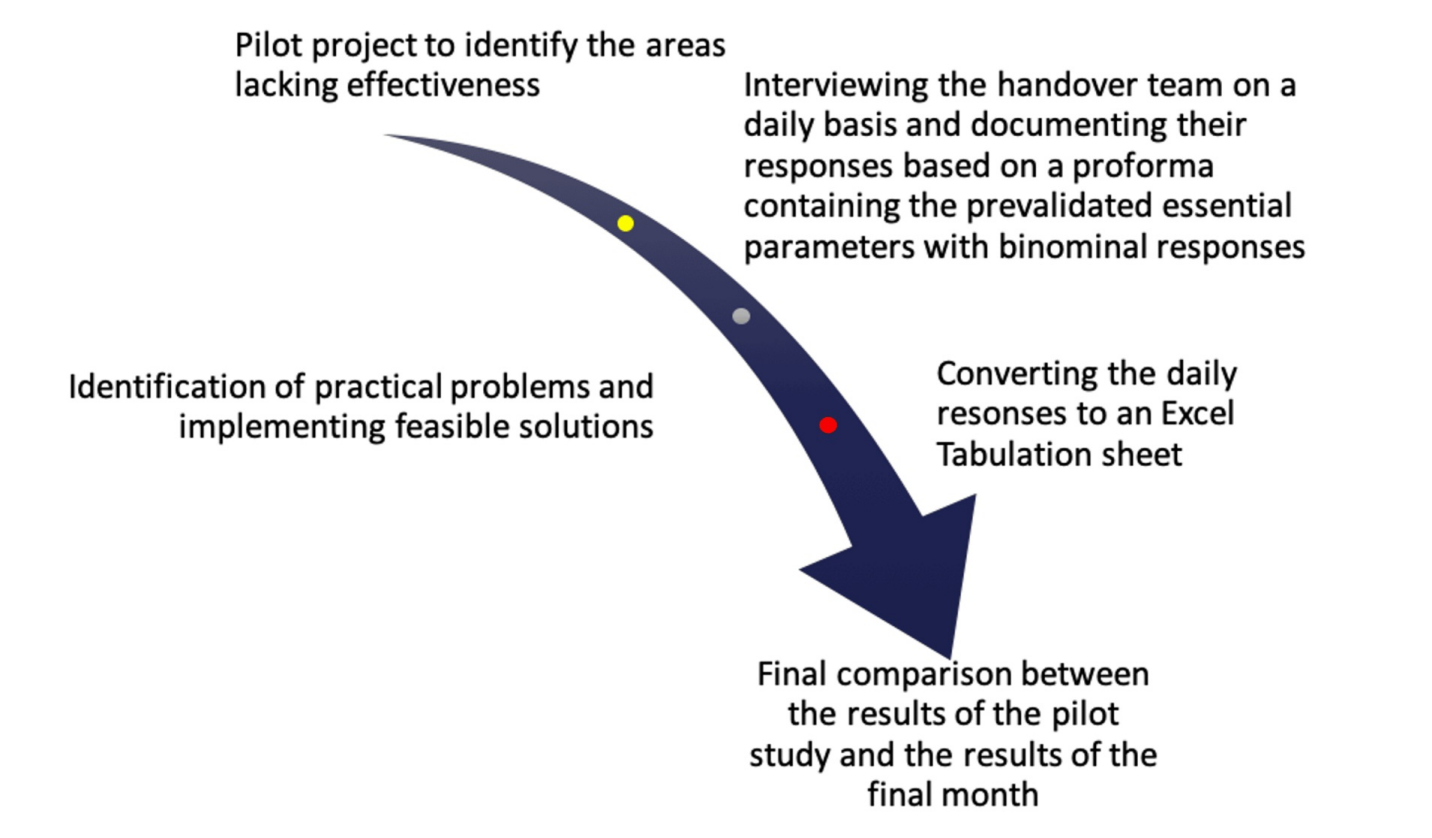
Structure of the Project

The structure of the project is given in Figure 1.

Since the focus was more on measuring the improvement, a statistical test was performed at the end of the study which could determine whether the improvement was significant or not. This was more or less in the same trajectory as the study done by Sadiq et al., in which the growth of improvement in the handover accuracy was measured statistically rather than the target of achieving 100% at the end of the project [6].

Structuring of human resources

In the newly framed situation, the gynaecology handover was supposed to happen in the Early Pregnancy Room (EPU) 1 at 0800 hours every morning. The handover was supposed to be attended by the incoming gynaecology consultant of the day, the outgoing and incoming registrar, the outgoing and incoming senior house officer (SHO) and the gynaecology nurse for the day. It was a two-tier system on the night on call (i.e. one labour ward registrar and one gynaecology registrar); hence, the presence of the night registrar was mandatory unless there was an ongoing operation in the emergency list or a dual emergency happening in the labour ward at the same time during the handover. The outgoing team would print out a hard copy of the handover document saved on the computer hard drive which was supposed to cover all the aspects including patients to be handed over in SBAR format (Situation, Background, Assessment and Recommendation), sick patients, patients waiting for theatre, pending referrals.

Statistical analysis

The results of every month were compiled in an Excel sheet and the parameters were very closely monitored. The problems were identified and the impact of the solutions applied were noted as well. When all the parameters were compared between the start date and the end date statistically, seven out of 11 parameters showed significant statistical improvement when compared with the help of the Mcnemar test.

## Results

**Figure 2:**
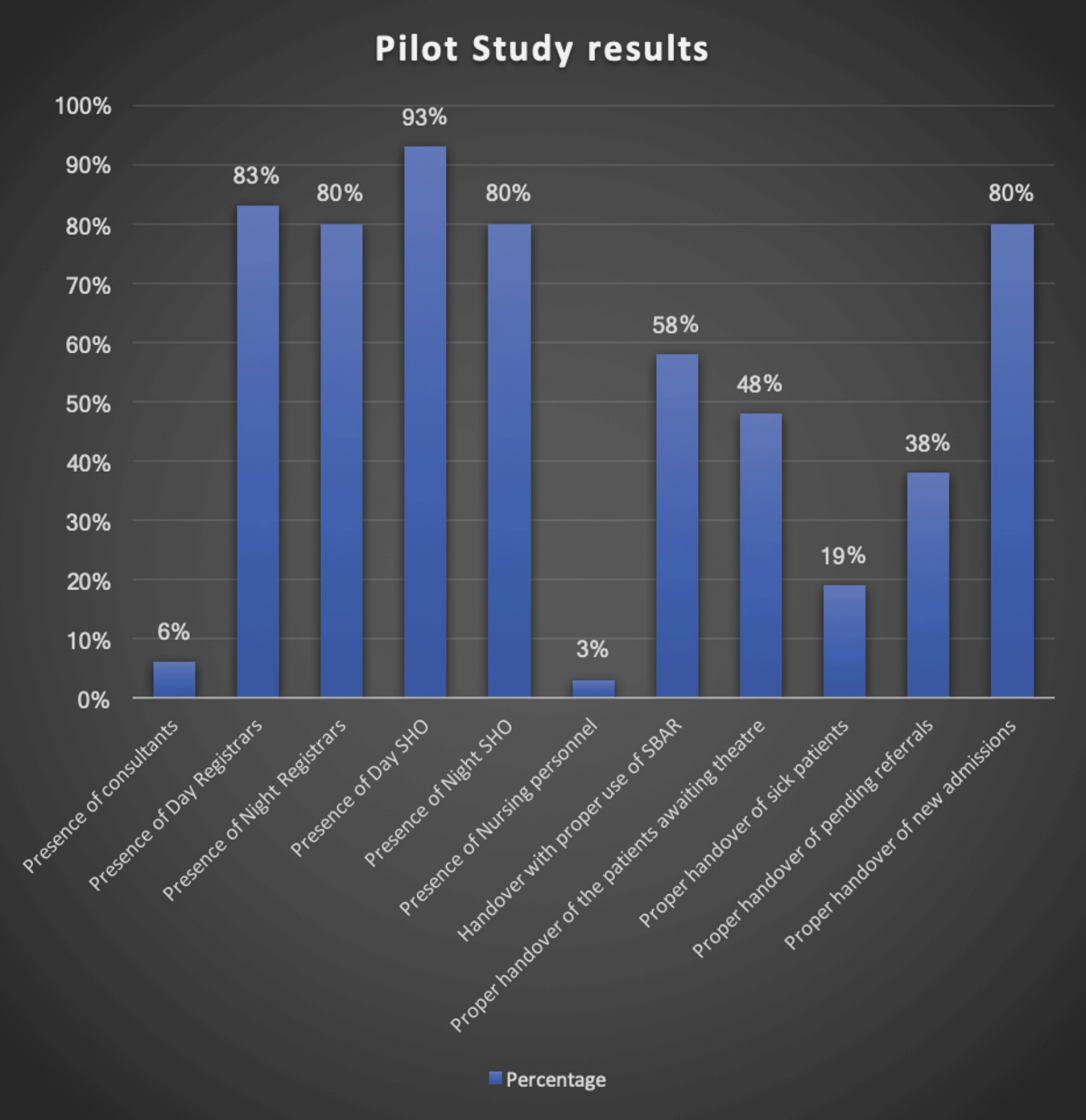
The outcome of the pilot study in a graphical demonstration SHO: Senior House Officer; SBAR: Situation, Background, Assessment, Recommendation

Before starting the project, a pilot study was done in the month of January on all the parameters and the result was plotted in a bar diagram (Figure 2). The study was done by interviewing the handover team after the actual handover and recording their responses. The results showed each and every parameter was recorded below the expected standard of 90% (Table 3). It was noted that these parameters would be compared with the final month parameter and the statistical significance would be noted.

**Table 3:** Result of the pilot study for the month of January SHO: Senior House Officer; SBAR: Situation, Background, Assessment, Recommendation; CEPOD: Confidential Enquiry into Peri-Operative Deaths

Parameters	Percentage
Presence of consultant	6%
Presence of day Registrar	83%
Presence of night Registrar	80%
Presence of day SHO	80%
Presence of night SHO	93%
Presence of nursing personnel	3%
Inpatients handed over in SBAR format	58%
Handing over of patients waiting for CEPOD theatre	48%
Sick/septic patients handed over	19%
Pending referrals handed over	38%
New admissions handed over	80%

The presence of the on-call gynaecology consultant was mandatory in the handover. Yet it was revealed in the pilot study that the presence of consultants was only 6% in the initial month. In the next five months, the team investigated the reason and applied some feasible solutions to increase their attendance rate. The result was followed over the next five months and the results were plotted in a graph which showed marked increase in the presence of consultants in the handover.

The presence of a night registrar was mandatory in the handover process. Ideally the second night registrar should be acting up as the gynaecology registrar for the night and should be present in the handover, the exception being an emergency situation going on in labour ward at the same time when the second registrar has been called as well or an ongoing CEPOD (Confidential Enquiry into Peri-Operative Deaths) case for which the registrar was occupied at that time. However, the pilot study revealed that the presence was around 80%. In the next five months, the team investigated the reason and applied some feasible solutions to increase their attendance rate. 

The day registrar should be present as a mandate during the process of morning handover. The exceptions being a scenario where the morning registrar needs to relieve the night registrar from the ongoing CEPOD case. In our study, the presence of the day registrar was found to be persistently more than the expected standard. Hence no intervention was needed to be applied in this parameter. The rota team was given positive feedback for their hard work in this regard.

The presence of day SHO was again compulsory as per the handover criteria. However, in our study this parameter always scored above the expected level hence no intervention was needed in this case. The rota team was given positive feedback for their hard work in this regard. The presence of night SHO was again compulsory as per the handover criteria. However, in our study this parameter always scored above the expected level hence no intervention was needed in this case. The rota team was given positive feedback for their hard work in this regard.

The presence of nursing personnel from the gynaecology emergency unit (GEU) was mandatory in the handover. However, the pilot study demonstrated the presence below expected standards. In the next five months, the team investigated the reason and applied some feasible solutions to increase their attendance rate. Table 4 describes the problems and their solutions.

**Table 4:** The parameters, their problems and solutions GEU: gynaecology emergency unit; SBAR: Situation, Background, Assessment, Recommendation; MEOWS: Modified Early Obstetric Warning Score

Parameters	Problem identified	Solutions
Presence of consultant	Consultants were not much aware of the new schedule and their job plans were not modified accordingly in time. Signatory sheets were not available for attendance during the handover.	Since the presence of Consultants were below the expected standards, the significant absence was escalated to the GEU lead. Handover attendance signatory sheets were created (Between February and March) and made available during the handover procedure Discussion about the topic in the consultants' meeting.
Presence of night registrars	The Night registrar shifts were not properly coordinated. Unavailability of 2^nd^ registrar in the night on-call rota.	Escalated to the Rota Lead and Rota Coordinator regarding the issue who corrected the rota gaps well covered in advance.
Presence of day registrars	The presence of the day registrar was always above the expected parameter, except in February	No advice was needed as the parameters were above the expected standard.
Presence of day senior house officers	The presence of the day senior house officer was always above the expected parameter, except in February	No advice was needed as the parameters were above the expected standard.
Presence of night senior house officer	The presence of the night senior house Officer was always above the expected parameter, except in February	No advice was needed as the parameters were above the expected standard.
Presence of GEU nursing staff	The presence of nursing personnel in the handover meeting was below standards There was an acute shortage of nursing staff in the rota.	Escalated the concern to the GEU lead for the absence of nursing personnel in handover who discussed the matter in an internal meeting. Escalated the concern to the Clinical Director regarding the lack of staff who recruited new nursing staff.
Pending referral handover	Not all the staff involved in the handover were added to the shared mailbox which was being used for gynaecology referrals. A considerable number of other disciplines were unaware of the correct email address for the mailbox used for gynaecology referrals,	All the members of staff who are involved in the process were added to the shared mailbox so that everybody can have unhindered access 24 x 7. All the other departments were mailed with the information regarding the Gynaecology Referral Mailbox which was to be used for any patient referral purpose.
Handover of new admissions	The handover of new patients were always above the expected parameter, except in February	No advice was needed as the parameters were above the expected standard.
Handover maintained in SBAR format	The team handing over the patients were not aware of the proper SBAR format	Departmental teaching was organized on the topic of SBAR handover where panel discussion was held on the same topic. Regular update sessions were organized in the department (both one-to-one and group session) on the same topic.
Handover of sick patients (with MEWOS score > 4 or 3 in any single parameter)	The team was not highlighting sick/septic patients during the handover	Discussed in internal meeting to highlight sick patients at handover Escalated to the GEU nurse to double check during the morning handover whether any sick/septic patient has been missed or not. A coloured highlighter was arranged and kept available to use in the handover room.
Handover of patients awaiting theatre	The patients awaiting theatre we’re not reviewed the night before theatre - their investigations were not up to date	Discussed in an internal meeting where a checklist was devised regarding the patients' waiting for the theatre. The checklist was supposed to be completed by the night team before the morning handover.

The referrals (both internal and external) were supposed to be collected in a generic mailbox and every clinician in the gynaecology department was supposed to have access to this mailbox. Ideally, every referral should have been addressed within 24 hours. Any referrals which have been received but not addressed in that particular shift would have been deemed as a pending referral. This pending referral should have been handed over to the next team during the gynaecology handover. In our pilot study, it was revealed that the performance regarding this matter was below the expected standards. In the next five months, focus was to identify the cause and apply feasible solutions.

The new admissions in the gynaecology ward were supposed to be included in the handover list during the morning handover. These patients would need follow-up plans from the day consultant. It was revealed in our study that this parameter was always above the expected standards. Hence no intervention was needed in regards to this parameter in the next five months. SBAR is an internationally accepted format in handover scenarios. The recommendation was every patient handover should follow SBAR protocol. In our pilot study, it was revealed that the performance regarding this matter was below the expected standards. In the next five months, the focus was to identify the cause and apply feasible solutions.

Any patient admitted to the ward with a MEWOS score of > 4 or 3 in any single parameter would be classified as sick/septic as per local protocol. These patients were supposed to be highlighted during the gynaecology handover by the team handing over. The most feasible way was to mark these patients with a coloured highlighter in the handover hard copy. In our pilot study, it was revealed that the performance regarding this matter was below the expected standards. In the next five months, the focus was to identify the cause and apply feasible solutions.

The patients who are booked for the morning CEPOD list are supposed to be handed over by the outgoing team to the incoming team with all the up-to-date investigation results. In our pilot study, it was revealed that the performance regarding this matter was below the expected standards. In the next five months, the focus was to identify the cause and apply feasible solutions.

**Figure 3:**
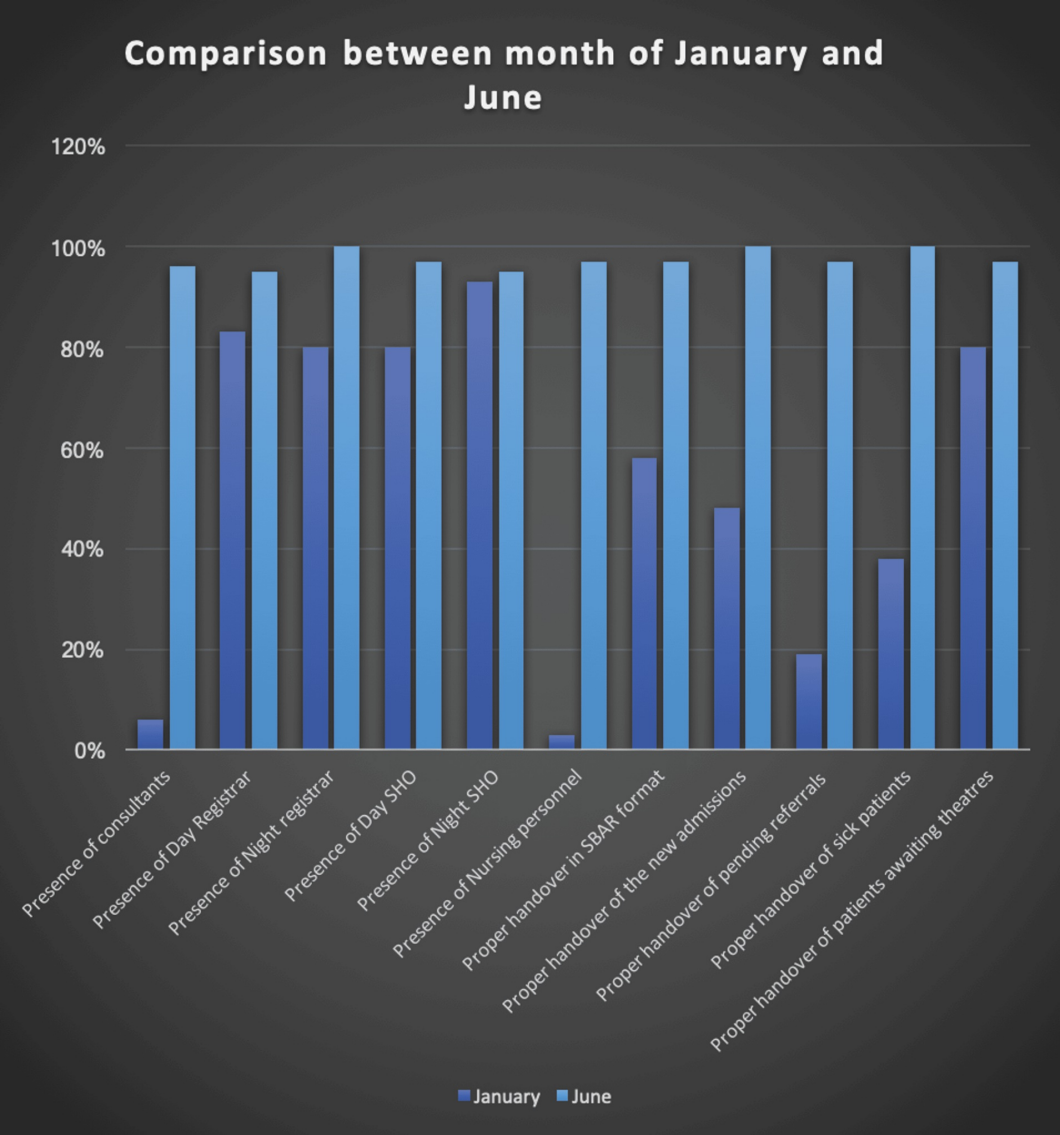
Graphical representation of comparison between January and June results SHO: Senior House Officer; SBAR: Situation, Background, Assessment, Recommendation

The results of every month were compiled in an Excel sheet and the parameters were very closely monitored. The problems were identified and the impact of the solutions applied were noted as well. When all the parameters were compared between January and June 2022 statistically, seven out of 11 parameters showed significant statistical improvement when compared with the help of the Mcnemar test. (Table 5). The results were also plotted in a Bar diagram as illustrated in Figure 3.

**Table 5:** Comparison between January and June statistics SHO: Senior House Officer; SBAR: Situation, Background, Assessment

Parameters	P-value
Presence of consultant	<0.05
Presence of day registrar	.89
Presence of night registrar	1.12
Presence of day SHO	1.2
Presence of night SHO	1.7
Presence of nursing personnel	<0.05
Inpatients handed over in SBAR format	<0.05
New admissions handed over	<0.05
Pending referrals handed over	<0.05
Sick patients handed over	<0.05
Patients awaiting theatre handed over	<0.05

## Discussion

The most extensive study on this matter was perhaps done by Alem et al. who demonstrated that handover is a complex process and that even basic information tools for handover support may have a significant effect [7]. In their opinion, solutions for facilitating medical handover should be thoughtfully created to prevent the weakening of some complicated handover functions that could result in subpar clinical outcomes. A well-researched and thoughtfully built tool, however, has the potential to reduce information failure, a significant reason for medical errors. In another study done by Behara et al., a few interesting facts about handovers were examined. In their study, it was concluded that handovers are much more than just communication drills. They are essentially exercises in developing distributed cognition since they include the transfer of authority, responsibility, and information through a process of co-construction in which both the incoming and departing parties are active participants. Future treatments may benefit from a deeper comprehension of the nature of handoffs in the medical field [8].

Hopkinson published her landmark study in 2002 [9]. In their study, it was detailed that for nurses working in NHS acute hospital wards, the nursing handover is a crucial task. The use nurses make of the information shared during nursing handover and the potential effects of certain nursing handover elements on patient outcomes have received little scholarly consideration. Data from a phenomenological study of 28 certified diplomate nurses were used for their study. Further research on the role of the nursing handover in providing emotional support for nurses could be fruitful, particularly if it could be linked to patient experiences and outcomes [9]. The study by Lally focused on the inter-shift handover between the nurses and the impact of communication in these scenarios. The inference was that the goal of the inter-shift handover nursing ritual is to strengthen the shared values among nurses. So, it shouldn't be viewed as a disused form of communication [10].

The study by Spooner et al. mainly involved a theme to install and assess a knowledge-to-action framework-based electronic minimum dataset for nursing team leader shift-to-shift handover in the critical care unit [11]. The results revealed team leaders used the electronic minimal dataset for handover in general (86%, n=49), and patient plan communication increased. However, important content pieces were missing from handovers, and doing the handover required more paperwork in addition to the required minimum dataset. The electronic minimal dataset was viewed more favourably by team leaders receiving handoff than by team leaders giving handoff among the 35 team leaders surveyed (n=35). The patient content (48%) and compatibility for short-stay patients (16%), as well as the tool's printing (12%), were all advantages of employing the electronic minimal dataset. However, almost half of the participants felt that the minimum dataset contained irrelevant information, had trouble navigating and finding pertinent information, and was missing pertinent information. The electronic handover interface was modified in response to suggestions for improvement. [11]

An interesting study on this aspect was conducted by Ghosh et al. in India [12]. This study concluded that in order to maintain continuity in patient care, the handover procedure is a crucial component of clinical practice on a daily basis. Standardizing clinical handover could lower sentinel events brought on by inefficient and imprecise communication. To determine the impact of implementing the SBAR protocol on the entire bedside nursing handover procedure, patient satisfaction, and nurses' acceptability, a single-arm experimental trial was conducted. All nursing staff on the designated unit, all handover procedures carried out by them, and patients admitted throughout the study period were included as a sample. An organized observation checklist was first used to evaluate the current handover procedure and patient satisfaction with nursing handover. Nursing staff received SBAR handover procedure training during the deployment phase. Following implementation, nursing handovers were once more evaluated, and information on patient satisfaction and nurses' acceptance was gathered. Between the prior and post-intervention groups, there was a statistically significant difference (P < 0.05) in the median scores for overall nursing handover and patient satisfaction with nursing handover. The nurse handover process can be improved, along with patient satisfaction and acceptance among medical personnel, by standardizing it [12].

In the study done by Bukoh and Siah in 2019, a Cochrane database review was published [13]. A search across six electronic databases, including Cochrane Central Register of Controlled Trials (CENTRAL), MEDLINE (Medical Literature Analysis and Retrieval System Online), CINAHL (Cumulated Index to Nursing and Allied Health Literature), Web of Science, Embase, and Scopus via Ovid summarized nine papers using the Cochrane Handbook for Systematic Reviews of Interventions and two independent reviewers. The conduct of this review and meta-analysis was guided by the Preferred Reporting Items for Systematic Reviews and Meta-Analyses statement. In their review of all studies published up through February 2019, it was concluded that current structured handover formats were effective in reducing problematic handovers such as omission of information, inaccurate information and documentation errors. The current study emphasized instrumenting a pre-validated proforma which can be highly useful while transferring information between the teams involved.

Another quality improvement project was launched by Tobiano et al. in which the aim was to improve intradepartmental handover. The project focussed on co-production and prototyping that took place during the intervention. The Handover Assessment Scale was used to gauge the nurses' perception of the handover's quality while observing handovers for intervention adherence. The study concluded that the project sparked ongoing development efforts that were required to continue meeting the demands of nurses who work across boundaries [14].

It has been hence clear that a comprehensive structure is very much essential for a successful handover process. It can only be achieved through proper planning and strategy with effective utilisation of the effective resources present in the department.

The study was designed at par with the prerequisites for a successful clinical handover. Every variable was selected and matched with the individual characteristic which must be present in the clinical handover between two teams. If we look at the pilot study, the presence of consultants in the handover process was only 6% which increased to 90% once the interventions were applied. This finding is aligned with the study done by Farhan et al. in which the mandatory presence of consultants in the handover was reinforced along with the guideline laid down by the Royal College of Physicians [15]. Regarding the presence of day and night registrar and day and night SHO, the percentage was already above the expected range; hence, no interventions were needed in this aspect. This was again reinforced in the quality improvement project published by Sadiq et al. in which they mentioned the importance of the correct mix of human resources in order to make the handover process to be successful [6]. According to the study done by Segal et al. on a systematic review of postoperative handover, it was also concluded that the presence of a correct mix of staff members is essential for a successful handover [16].

Regarding the presence of GEU nursing staff, the presence was below standard in the pilot study done in January at 3%. After applying proper interventions, the presence increased to 97%. This was again in alignment with the study done by Chien et al., which mentioned the success of the change process depended on strong leadership to advocate for change, continual mentoring and reinforcement of new practices, and collaboration with nurses [17]. Next, when we focused on the pending referral handover, it was revealed that only 38% of the pending referrals were getting handed over properly in January, which increased to 90% in June. The study by Robertson actually noted a 13% error in information transfer which is a serious hazard to patient safety [18].

According to the study done by Shahrami et al., it was recommended that a structured proforma can be useful in covering up these missing links [19]. However, in our project, it was seen that a shared mailbox folder was found to be extremely beneficial in improving this parameter. This fact is also supported by the landmark study done by Robertson et al., in which they compared 29 studies (two randomised control studies and 27 non-randomised control trials) and concluded that there is no single methodology which can be declared as the gold standard as the method of handover. Hence it was recommended that the methodology needs to be individualised to suit the requirements of the particular unit concerned.

Regarding the proper handover of inpatients in SBAR format, we could see a marked rise from 58% in January to 97% in June after the application of proper interventions in the meantime. The SBAR handovers are of paramount importance in the modern day-to-day handover process as described by Ruhomauly et al [20]. In this study, it was found that only 54% of the nurses were using the SBAR format in a district general hospital in the UK though 100% of the nurses were aware of it. They also mention that visual aids and ward-based education sessions may provide efficient and scalable ways to raise knowledge and comprehension of the SBAR communication tool for handovers. In order to improve communication, top staff employees must be encouraged to support a positive handoff culture [20]. In our hospital, the situation was somehow similar as the concept of SBAR was present but was not emphasised enough to reap the results. In our project, the SBAR handover teachings were organised where several incidents were cited in which non-implementation of SBAR led to serious incidents. The teachings were well attended and well recognised. The reflection was evident in the gradual rise in the rates of patients handed over in the SBAR format.

The percentage of proper handover of new admissions was 89% in January which was 1% short of the desired level of 90%. On subsequent audits of the next few months, the level was always consistently above 90%; hence, this parameter did not need any intervention as it was above the desired level to achieve patient safety. It is also mentioned in the study done by Din et al. that a robust handover process is essential for patient safety and to avoid unwanted serious incidents [21].

Regarding the parameter of sick patients being handed over in the proper manner, in January the percentage was 19% which subsequently increased above 90% in the next six months after the application of appropriate interventions. This was again in alignment with the study done by Agha et al. where they concluded that ordering complicated clinical processes by structure, standardisation, and checklists increases productivity and safety [22]. Regarding the parameter of proper handover of the patients waiting for theatre the next day, the pilot study in January revealed that only 48% of these patients were handed over in a proper manner. However, with proper intervention, this rate increased to above 90% in the next six months. This was also been reflected in the study by Ah-Kye et al. where the introduction of a traffic light-coded system caused much improvement in the handover process [23]. At this point, it is worthy of mentioning the systematic review by Pucher et al. in which the effectiveness of interventions employed during a handover was analysed [24]. It was concluded that standardised operating protocols could lead to potential improvement in the handover process; however, more research is needed in this field.

Limitations

The study was limited by its small number of subjects. The results lacked external validity as the problems of each department could be different and thereby the solutions will change as well. The entire study was based on the response generated by interviewing the team receiving the handover each day. The team would be composed of a variety of individuals and hence the response generated could be quite subjective. Also, any permanent change of practice can only be confirmed with a long-term follow-up of the subject. In our study, the follow-up was only for six months during which the corrective measures were applied. Thus, it is uncertain whether the solutions would survive the test of time and prove to be useful in the long run. SHOs not previously familiar with gynaecology and obstetrics staff would improve clinically in the speciality in the time frame described, becoming more familiar with the system and the hospital handover may have improved over time due to this increase in skill level rather than our interventions. Also, the study did not include the impact on services (that is, reduction in near-miss cases and deaths) due to the improvement in parameters. The parameters measured are interdependent; hence, it was difficult to objectify which solution is the true driving force behind the improvement of a typical parameter. This would have been an ideal measure of the holistic gains achieved by the project. We do aim to cover that topic in a much wider study later on.

## Conclusions

A structured multidisciplinary team handover is the core component of continuity of care and patient safety. In this quality improvement project, we aimed to study the system which existed already to find out its limitations and to provide practical solutions aimed at substantial improvements. In order to investigate the understanding of what happened in the department on the previous day, we looked into all information throughout the course of the entire five months, either episodically or methodically. Not all of our handover process interventions (i.e., information tools) were effectively deployed; in particular, the information-yielding tools with the highest yield potential were not.

Our research demonstrates that handover is a complex process and that even basic information tools for handover support may have a significant effect. In our opinion, solutions for facilitating medical handover should be thoughtfully created to prevent weakening some complicated handover functions that could result in subpar clinical outcomes. A well-researched and thoughtfully built tool, however, has the potential to reduce information failure, a significant reason for medical errors. We tried to demonstrate that once we find the source of an issue by doing a root cause analysis, it is indeed feasible to provide a practical solution that will improve the quality of that practical parameter. However, this requires continuous audits to reconfigure the solutions according to the practical needs and develop solutions to new problems. This continuous scrutiny is hugely dependent on human resource availability. In our study, we did find a statistically significant improvement in implementing the simple yet effective and practically feasible solutions which again reinforces the simple fact of root cause analysis and finding solutions according to the need and resources.

## Appendices

Daily handover proforma

Date -

Time -

A.    Team Presence

1.     Presence of consultant - Yes/No

2.     Presence of Night Registrar - Yes/No

3.     Presence of Night SHO - Yes/No

4.     Presence of GEU Nursing staff - Yes/No

5.     Presence of Day SHO - Yes/No

6.     Presence of Day Registrar - Yes/No

B.    Inpatients handed over in SBAR format - Yes/No

C.     Sick/Septic patients handed over - Yes/No

D.    Patients awaiting CEPOD theatre handed over - Yes/No

E.     Pending referrals handed over - Yes/No

F.     New admissions handed over - Yes/No

If No to any answer, the reason behind:

________________________________________________________________________________________________________________________________________________________________________________________________________________________________
